# Printing 2-Dimentional Droplet Array for Single-Cell Reverse Transcription Quantitative PCR Assay with a Microfluidic Robot

**DOI:** 10.1038/srep09551

**Published:** 2015-04-01

**Authors:** Ying Zhu, Yun-Xia Zhang, Wen-Wen Liu, Yan Ma, Qun Fang, Bo Yao

**Affiliations:** 1 Institute of Microanalytical Systems, Department of Chemistry, Zhejiang University, Hangzhou, 310058, China

## Abstract

This paper describes a nanoliter droplet array-based single-cell reverse transcription quantitative PCR (RT-qPCR) assay method for quantifying gene expression in individual cells. By sequentially printing nanoliter-scale droplets on microchip using a microfluidic robot, all liquid-handling operations including cell encapsulation, lysis, reverse transcription, and quantitative PCR with real-time fluorescence detection, can be automatically achieved. The inhibition effect of cell suspension buffer on RT-PCR assay was comprehensively studied to achieve high-sensitivity gene quantification. The present system was applied in the quantitative measurement of expression level of mir-122 in single Huh-7 cells. A wide distribution of mir-122 expression in single cells from 3061 copies/cell to 79998 copies/cell was observed, showing a high level of cell heterogeneity. With the advantages of full-automation in liquid-handling, simple system structure, and flexibility in achieving multi-step operations, the present method provides a novel liquid-handling mode for single cell gene expression analysis, and has significant potentials in transcriptional identification and rare cell analysis.

Understanding the functions and behaviors of cells in various physiological environments is the basic task of cell biology research. Although all cells in an individual organism have almost identical genotype, the gene expression variations in these cells generate diverse cell types with unique functions and behaviors. Nowadays, most of our knowledge on biological process is on the basis of the study of large populations of cells, which limits the in-depth understanding of cell differentiation, the sensitive diagnosis of major diseases, and the reliable analysis of rare cells^1^,^2^,^3^,^4^. Single-cell reverse transcription polymerase chain reaction (RT-PCR) is a powerful tool for the measurement of gene expression variation among individual cells, which has successfully applied in the study of cell heterogeneity of human and mouse stem cells^5^,^6^, gene expression dynamics of cells from early development stage of mouse embryo^7^, and gene expression signatures that are relevant to patient survival and clinical outcome in colon cancer patients^8^. However, conventional techniques for single-cell RT-PCR assay, where single cells are commonly handled using micropipettes under microscopes and RT-PCR assays are performed in PCR tubes, are cumbersome, low throughput, high reagent consumption, and relatively low sensitivity.

Microfluidic systems provide a promising and practical platform for single cell RT-PCR assay. Single cells can be reliably isolated and manipulated using microfabricated channels, valves^6^,^9^, or grooves^10^. Confining PCR reactions in ultra-small volumes can reduce the reagent consumption, and significantly increase the sensitivity of PCR assay with a limit of detection of single molecule^11^,^12^. Microfluidics techniques also offer the abilities of parallel analysis^6^,^8^,^11^, automated operations^13^, and multi-step integration on single chip^14^,^15^. Toriello et al.^14^ developed an integrated single-cell gene expression analysis device capable of performing single cell capture, cell lysis, reverse-transcription of the released mRNA to cDNA, PCR amplification of the cDNA, and quantification of the PCR product with capillary electrophoresis. To achieve direct quantification of single-cell gene expression and increase analysis throughput, White et al.^15^ combined two-step reverse transcription quantitative polymerase chain reaction (RT-qPCR) with parallel liquid operation using arrays of microchambers and microvalves. All steps including cell trapping in a specially-designed microgrooves, cell lysis by heating, cDNA synthesis, PCR amplification, and real time fluorescence detection were integrated on a single chip. Real time PCR (qPCR) enabled the direct quantification of gene copies by detecting the fluorescence intensity of PCR solutions at each thermal cycle and obtaining their threshold cycles (Ct). In a recent work, the same group further applied digital PCR technique in single-cell RT-PCR assay to achieve absolute measurement of gene targets^16^.

Besides microvalve and microchamber-based systems, droplet-based microfluidic systems provide another efficient way for single-cell RT-qPCR assay. Droplet-based microfluidic systems usually use microdevices to generate and manipulate picoliter to nanoliter-scale water-in-oil droplets^17^,^18^. Each droplet can be regarded as a “virtual test tube” that can perform miniaturized chemical or biological reaction without cross contamination. Compartmentalizing the aqueous droplet with oil phase can also eliminate liquid evaporation, avoid sample absorption on solid channel surface, provide biocompatible environments for enzyme reactions, and enhance heat transfer in PCR assay^19^,^20^. Single molecule amplification can be implemented in droplet format with high efficiency and high throughput^21^,^22^,^23^. For single cell RT-PCR assay, usually droplets containing single cells and PCR reagents are rapidly generated in T-junction^24^ or flow-focusing junction channels^25^,^26^. After the cells are lysed with chemicals or heating, the droplets are collected into PCR tubes to perform RT-PCR assays on routine thermal cyclers. Finally, the fluorescence intensities in droplets are measured with flow cytometry or fluorescence microscopy to obtain the gene expression levels in single cells^24^,^25^. With the advantages of high throughput and high sensitivity, these droplet systems are suitable for large-scale comparatively profiling gene expression differences in different cell lines or tissues, while may not be suitable for precise real-time quantifying of the gene expression in small pools of cells. Mary et al.^26^ developed a droplet-based single-cell RT-qPCR system by storing droplets in microchannels and collecting fluorescence images at each thermal cycle. Unfortunately, the numbers of gene copy in single cells were not obtained due to the lack of standard curves from exponentially diluted samples. In addition, these droplets were movable during the thermocycling process, making it challenging to accurately record the fluorescence changing of large number of droplets.

In this paper, we present a different type of droplet system capable of achieving single-cell RT-qPCR assay by printing a 2-dimentional droplet array on an oil-covered microchip with a microfluidic robot^27^,^28^. The microchip was patterned with hydrophilic spots to immobilize the printed droplets for subsequent droplet manipulation and PCR reaction. The microfluidic robot was built mainly on the basis of our previously-developed sequential operation droplet array (SODA) system^28^,^29^. The SODA system is capable of performing automated and flexible droplet generation and manipulation with variable droplet volumes from 60 pL to 200 nL, and has been applied in screening of inhibitors from a chemical library^28^, cell-based drug combination study^29^, and protein crystallization^30^. In the present work, we integrated all the operations required in single cell RT-qPCR assay on a droplet array system including single cell encapsulation, cell lysis, reverse transcription, and quantitative PCR with real-time fluorescence detection, which provides a novel liquid-handling mode for single cell gene expression analysis. The feasibility of the present system for single cell gene expression analysis was demonstrated in quantitative measurement of microRNAs, which plays important roles in gene regulation^31^, cell proliferation, and disease development^32^.

## Results

### System design

**Figure 1:**
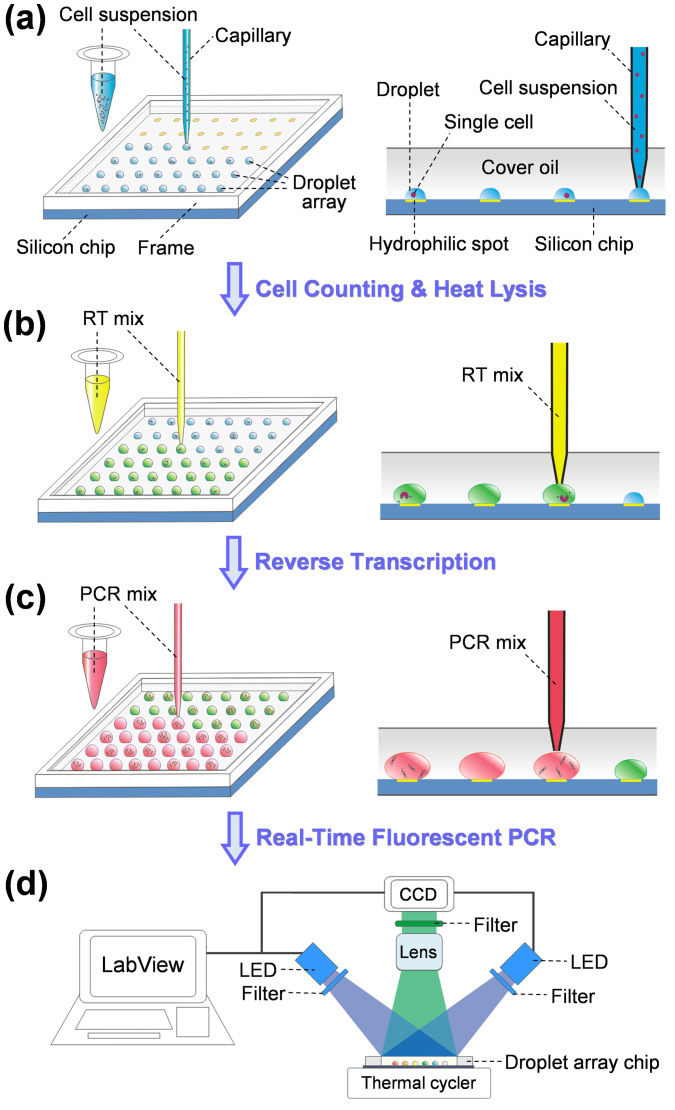
Schematic diagrams showing the operation procedures of droplet array system for single cell gene expression analysis. (a) Cell suspension is printed on the hydrophilic spots of silicon chip to generate droplet array containing cells; (b) After counting cell numbers in droplets and thermally lysing the cells, reverse transcription mix is continuously added into each droplet to convert RNA to cDNA; (c) PCR mix is added into each droplet followed by (d) real-time fluorescence PCR to quantify the gene expression levels.

In the present droplet array system, droplets are covered with a layer of mineral oil to eliminate droplet evaporation. Such a semi-open feature of the droplet array can substantially facilitate the multi-step droplet manipulations required in single cell RT-qPCR assay including droplet generation, single cell encapsulation, cell lysis, reagent addition, reverse transcription, and quantitative PCR by allowing the capillary probe of the microfluidic robot to directly contact with droplets through the cover oil (Figure 1).

The reaction inhibition of RT-PCR by cell lysate and buffer is a major challenge for the direct quantification of gene expression in single cells. Such inhibition effects can be efficiently mitigated by diluting the cell lysate with RT and PCR reagents^15^,^24^. Taking the advantage of the present system in varying the droplet volumes, the PCR reaction was performed in a droplet volume of 50 nL, including 2-nL cell lysate, 18-nL RT reagent, and 30-nL PCR reagent, ensuring a dilution factor of 50:2 (PCR reagent : cell lysate). The total PCR reaction volume of 50 nL was chosen by compromising the density of droplet array, stability of droplets, and sensitivity of fluorescence detection. Cell suspension buffer was also optimized to further reduce the reaction inhibition effect as detailed description in the section of “*Inhibition of cell suspension buffer on RT-PCR*”.

In droplet-based single cell analysis, usually the cell suspension is required to be extensively distributed into large number of small-volume droplets, and multiple reagents are required to be added in these droplets. The previous SODA system was operated with a one-by-one mode, under which each droplet reactor was generated by sequentially assembling sample and reagents into capillary probe, and then deposited it on microchip^28^. Such an operation mode is particularly suitable for dealing with droplets of multiple different samples, such as in screening of enzyme inhibitors from a chemical library^28^, which would significantly limit the droplet processing throughput when applied in single cell analysis due to the repetitive aspirating, depositing, and long-distance moving between sample/reagent reservoirs and microchip. Thus, to increase droplet processing speed, in this work, the droplet generation and reagent addition operations were performed in a mode of continuous-printing, where large volume of sample/reagent solution was firstly aspirated into the capillary and then continuously dispensed on the hydrophilic spots or into the preformed droplets on the chip with a definite volume for each spot/droplet (See Figure 1). With the continuous-printing method, the repetitive aspirating and long-distance moving steps were significantly reduced. Compared with the time consumption of over 30 min in the previous SODA system with one-by-one mode^28^, 100 droplets with droplet volume of 2 nL could be generated within 5 min.

One concern of using the microfluidic robot in RT-qPCR assay is the possible cross contamination between droplets due to the repetitive droplet manipulation with single probe. We adopted two measures to minimize the possible cross contamination. A surfactant (Span 80) was added into carrier oil (0.5%, v/v) in the capillary probe to improve the wetting ability of carrier oil on the inner surface of the capillary. The improved wetting ability could create a steady layer of oil between aqueous sample droplets and the channel surface^28^,^33^. The oil layer significantly prevented the interaction of nucleic acids and proteins with channel surface, and thus reduced possible cross contamination^33^. The other efficient way is to wash the capillary probe thoroughly by sequentially using RNase-free ethanol, 2% RNase Remover, and DEPC-treated water, while handling different RNA samples and reagents. We tested the cross contamination between alternatively generated droplets containing mir-122 of 9.6 × 10^8^ copies/droplet and DEPC-treated water by measuring their Ct values and calculating their concentrations (Figure S1). The average concentration of mir-122 in the contaminated DEPC-treated water droplets was 2.12 × 10^5^ ± 1.8 × 10^5^ copies/droplet (n = 3), indicating the cross contamination is 1/4528 of sample droplets. When washing steps were added, the average Ct value of the DEPC-treated water droplets was 55 ± 23 copies/droplet (n = 3), demonstrating the cross contamination can be effectively reduced by three orders of magnitude using the washing steps. Thus, the washing step was employed in the present work while using the probe to deal with different samples and reagents.

### Performance for RT-qPCR assay

**Figure 2:**
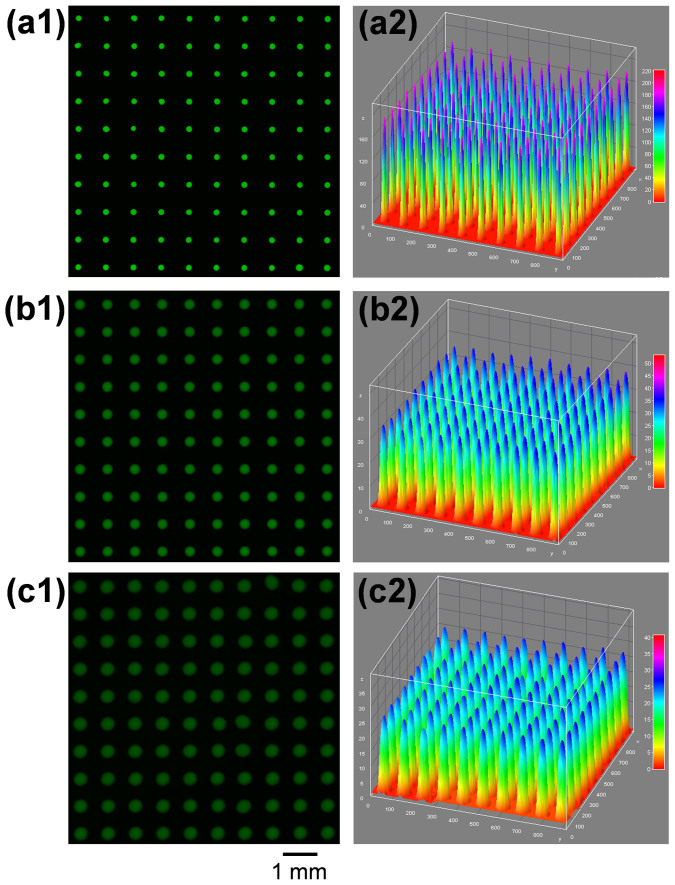
Evaluation of the performance of the droplet robot in droplet manipulation under continuous-printing mode. (a) A 10 × 10 array of 2-nL droplets with sodium fluorescein as model sample (100 μM). (b) 10 × 10 array of 20-nL droplets generated by continuously dispensing 18 nL of 10 μM fluorescein solution into each droplet in droplet array (a). (c) 10 × 10 array of 50-nL droplets generated by continuously dispensing 30 nL of 10 μM fluorescein solution into each droplet in droplet array (b).

For RT-qPCR assay, the droplet robot was operated under continuous-printing mode, under which sample and reagent solutions were continuously printed on the microchip with three different volumes of 2 nL, 18 nL, and 30 nL. To evaluate the performance of the droplet robot in droplet manipulation, we firstly generated a 10 × 10 uniform array of 2-nL droplets using sodium fluorescein (100 μM) as a model sample (Figure 2a), and then continuously dispensed 18 nL low-concentration sample solution (10 μM) into each droplet on the array to form a 20 nL droplet array (Figure 2b), and at last dispensed 30 nL of 10 μM fluorescein solution into each droplet of the 20 nL droplet array, forming a 50 nL droplet array (Figure 2c). The fluorescence intensities of the droplets in the three arrays were measured and their relative standard deviations (RSDs) were calculated as 2.68%, 2.73%, and 2.67% (n = 100), respectively. The low RSDs demonstrated the high precision and reliability of the droplet robot in multiple-volume droplet manipulation under the continuous-printing mode.

**Figure 3:**
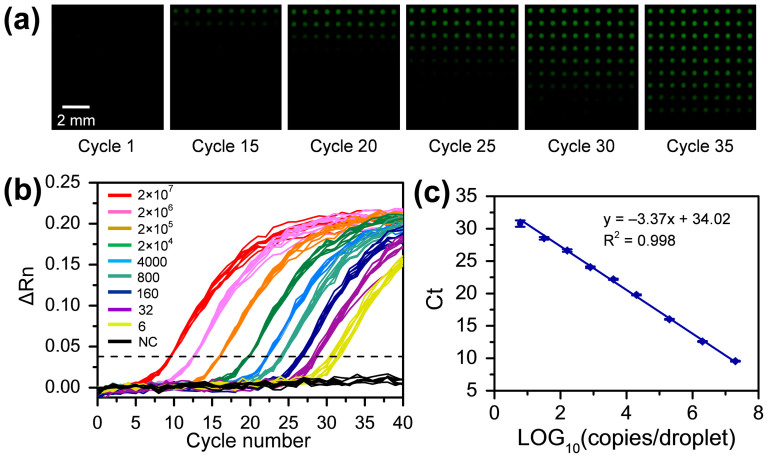
Evaluation of the sensitivity and reproducibility of the nanoliter droplet array system for RT-qPCR assay by measuring synthetic mir-122 samples with a wide range of concentrations (from 6 to 2 × 10^7^ copies/droplet). (a) Fluorescent images of droplet array containing different sample concentrations recorded at different thermal cycles. The input of mir-122 is 0, 6, 32, 160, 800, 4000, 2 × 10^4^, 2 × 10^5^, 2 × 10^6^ and 2 × 10^7 ^copies/droplet from the bottom row to the top row, respectively; (b) Real-time amplification curves generated by processing the fluorescent images in (a). The dashed line is used to indicate the Ct values; (c) Standard curve of synthetic mir-122. Each droplet contains 2 nL mir-122 sample, 18 nL RT reagent, and 30 nL PCR reagent.

To evaluate the sensitivity and reproducibility of the nanoliter droplet array system for RT-qPCR assay, synthetic mir-122 samples with a wide range of concentrations (from 6 to 2 × 10^7^ copies/droplet) in droplet array were measured. Each 50-nL droplet containing 2 nL sample, 18 nL RT reagent, and 30 nL PCR reagent was generated with three-step droplet printing method. The fluorescent images of the droplet array taken at different thermal cycles are shown in Figure 3a, and the corresponding real-time amplification curves are shown in Figure 3b. The linear fit of the logarithm of copy number of mir-122 template per droplet versus Ct value is shown in Figure 3c and an excellent linear relationship is obtained in the concentration range of 6 to 2 × 10^7^ copies/droplet (R^2^ = 0.998). The standard deviations (SDs) of the Ct values were 0.07, 0.06, 0.07, 0.09, 0.10, 0.13, 0.18, 0.20, and 0.49 (n = 10) for droplets with mir-122 concentrations of 2 × 10^7^, 2 × 10^6^, 2 × 10^5^, 2 × 10^4^, 4000, 800, 160, 32, and 6 copies/droplet, respectively. These results demonstrate the high uniformity of droplet array-based PCR amplification technique, which is comparable to the microvalve-based qPCR technique reported by White el al.^15^. The minimum amount of mir-122 template could be measured with the present RT-qPCR system was *ca.* 6 copies/droplet, which is much lower than the detection limit of 1000 copies/droplet in large-volume droplet system (500 nL)^27^. This result shows that the reduction of droplet volume not only reduces sample and reagent consumption, more importantly, but also improves PCR amplification efficiency (from 86.3% in 500-nL reaction system to 98.03% in 50-nL reaction system) and detection sensitivity. Such an improvement could be attributed to the enhanced heat transferring and the increased concentrations of low abundance genes within smaller volume droplets.

### Inhibition of cell suspension buffer on RT-PCR assay

**Figure 4:**
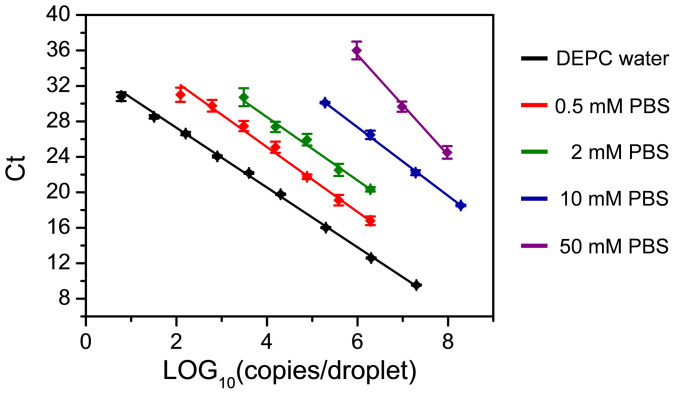
The inhibition effect of PBS buffer on PCR amplification efficiency. Standard curves are generated by exponentially diluting mir-122 samples using PBS buffer with different concentrations including 50 mM (5×), 10 mM (1×), 2 mM (0.2×), 0.5 mM (0.05×), and 0 mM (negative control, DEPC-treated water).

During cell encapsulation step, cells were suspended in a buffer solution containing salts and culture mediums. In the preliminary experiments, we observed this buffer solution produced significant inhibitory effect on the subsequent RT and PCR steps. Although this inhibitory effect could be mitigated by diluting the cell lysate with RT and PCR reagent, significant decreases of PCR efficiency and sensitivity were still observed. Thus, we further tested and optimized cell suspension buffer to find the optimal condition for single cell RT-PCR. We first investigated the inhibitory effects of PBS buffer (1×, 10 mM), DMEM medium (1×), and DMEM medium (1×) containing 10%FBS, respectively, by preparing synthetic mir-122 samples (1.92 × 10^8^ copies/droplet) with these buffers, measuring their Ct values, and comparing with the control sample prepared with DEPC-treated water. The Ct values for DEPC-treated water, PBS buffer (1×, 10 mM), DMEM medium (1×), and DMEM medium (1×) containing 10% FBS were 5.6, 6.5, 9.1, and 9.0, respectively. The PBS buffer has the lowest inhibitory effect on the RT-PCR amplification of mir-122. Then, we tested the effect of PBS concentrations on PCR efficiency and detection sensitivity by analyzing exponentially-diluted mir-122 samples prepared in PBS buffers. PBS buffers with five different concentrations including 50 mM (5×), 10 mM (1×), 2 mM (0.2×), 0.5 mM (0.05×), and 0 mM (negative control, DEPC-treated water), were tested, respectively. The linear fits of the logarithm of mir-122 copy number per droplet versus Ct values under these concentration conditions are as shown in Figure 4, and the PCR efficiencies and detection limits calculated from these curves are shown in Table 1. With the decrease of PBS concentration, the PCR efficiency was gradually increased from 50% at 50 mM to 98.04% at 0 μM, and the detection limit was significantly decreased from 1,000,000 copies/droplet at 50 mM to 6 copies/droplet at 0 μM, demonstrating the decrease of PBS concentration can efficiently reduce the inhibitory effect. However, one possible risk to use low concentration PBS buffer in cell suspension is the spontaneous cell lysis due to osmotic imbalance. We examined the stability and viability of cells suspended in diluted PBS buffers with concentrations of 2 mM (0.2×) and 500 μM (0.05×) by continuously observing the cell state under a microscope. The cells were quite stable in 2-mM PBS buffer without significant change in cell size in a period of 1 hour, while cell swelling and lysis were observed in 0.5 mM PBS buffer. We further investigated the effect of PBS buffer concentrations on microRNA integrity during cell handling and lysis steps. Huh-7 cells were suspended in two different PBS buffers (0.2× and 1×) and lysed at 95°C for 5 min, respectively. After cell lysis, the PBS concentrations in the two cell lysates were adjusted to be the same (0.2×) and then their Ct values were measured in 500-nL droplets with 10-cell equivalent lysate in each droplet. As shown in Figure S2, the average Ct values were calculated as 24.08 ± 0.13 (n = 10) and 24.19 ± 0.19 (n = 12) for 0.2× and 1× buffers, respectively. These results demonstrated the PBS buffer concentration has no evident effect on the microRNA integrity. Therefore, in single cell experiments, cells were suspended in 2-mM PBS buffer in the cell encapsulation and lysis steps to reduce the inhibitory effect of cell suspension buffer and increase the detection sensitivity of RT-PCR assay.

### Measurement of MirRNA Expression in Single Cells

**Figure 5:**
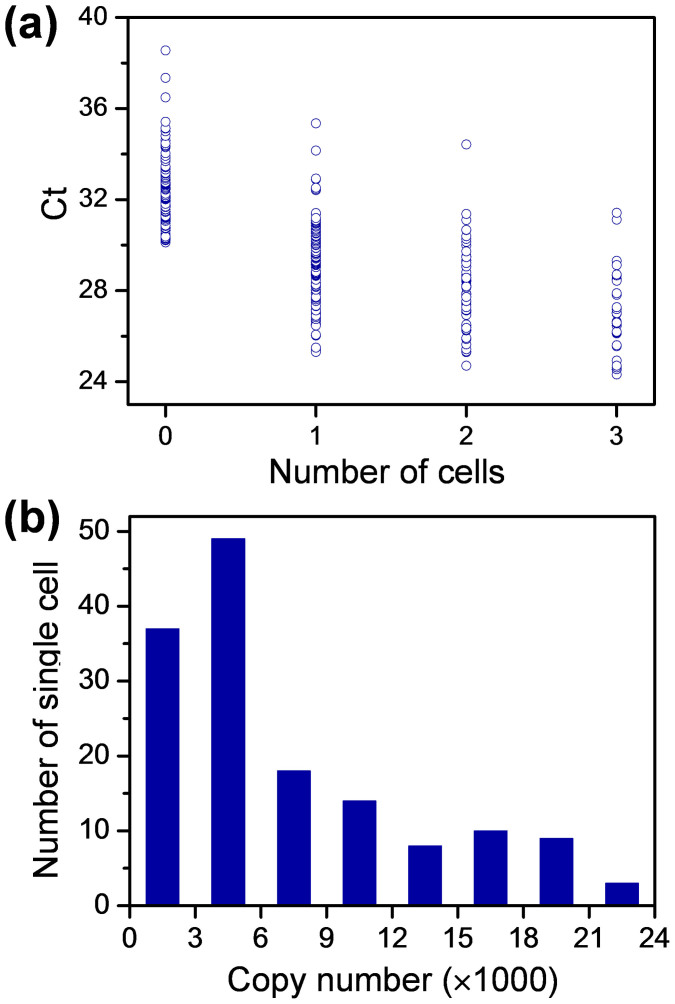
Mir-122 expression in single cells. (a) The Ct-value measurement for droplets containing 0, 1, 2, and 3 Huh-7 cells. Each droplet containing 2 nL cell suspension, 18 nL RT mix, and 30 nL PCR mix. (b) The distribution of mir-122 copy number in single Huh-7 cells.

We applied the droplet-based RT-qPCR assay system to study mir-122 expression in single Huh-7 cells. Mir-122 is a mammalian liver-specific microRNA, which is implicated as an important regulator of liver development, fatty-acid metabolism, and hepatitis C virus replication^34^. Huh-7 cells were chosen as the model sample because mir-122 is highly expressed in the cell lines. The cell suspension concentrations were firstly optimized to obtain highest single cell occupancy in droplet array. A highest probability of 35% for single cell encapsulation was achieved with a droplet volume of 2 nL and cell suspension concentration of 500,000 cells/mL. The cell number distribution in all the droplets shows good agreement with Poisson distribution (Figure S3). To demonstrate the feasibility and throughput of the present droplet array system for single cell analysis, total 360 droplets containing 126 single-cell droplets, 60 two-cell droplets, 30 three-cell droplets, and 144 empty droplets were measured. Figure 5a shows the Ct value distributions for the droplets containing single cell, two cells and three cells. The Ct values for empty droplets were all higher than 30.7, which were beyond the linear region of standard curve with RNA input from 3000 copies/droplet to 1,000,000 copies/droplet (Figure 4, PBS concentration of 2 mM). For the single-cell droplets, their Ct values had a relative broad distribution from 25.3 to 35.4 (Figure 5a). After the single-cell droplets with Ct values over 30.7 are removed, the copy number distribution of mir-122 is calculated as from 3061 to 79998, which indicates the highly variable mir-122 expression in Huh-7 cells. The mean copy number of mir-122 in single Huh-7 cells as 14525, which is comparable to the reported value of 16000 copies/cell^35^.

## Conclusion

In summary, we demonstrated RT-qPCR-based single cell gene expression analysis can be realized in droplet-based microfluidic form by printing nanoliter-scale droplets on a surface-modified silicon chip. Since the whole liquid handling operation is fully-automated and programmable, it can perform single cell gene expression analysis not only with two-step RT-qPCR protocol as used in this work, but also with other protocols (such as one-step RT-qPCR) as demanded. Compared with microvalve-based single cell RT-qPCR systems^14^,^15^, the present droplet array system holds the advantages of low cost, simple structure, and easiness of operation. In addition, the droplet array system with semi-open property can facilitate subsequent single cell gene analysis (such as gene sequencing) after PCR amplification by using the capillary probe to sample from PCR product droplets through oil layer. In addition, the silicon chip can be reused for over hundreds of times within several months with simple washing procedure by detergent, RNase remover, and DEPC-treated water. We envision the droplet array system could find broad applications where only limited samples are available, such as the study of differentiation process of embryonic stem cells, the identification of circulating tumor cells, and the sample preparation for single cell genome sequencing.

The analytical performance of the present system could be further improved in future work. The droplet processing speed can be linearly increased by using multiple capillary probes. The efficiency of single cell encapsulation in droplets can be improved by immobilizing single cells in specially-designed single-cell trapping microstructures^36^ or cell adhesion surfaces^14^,^37^ before droplet generation. Besides, immobilization of single cells can also facilitate to thoroughly wash them to minimize the background signals arising from free RNAs in the medium, which could be ascribed to active cell secretion or passive release after unexpected cell lysis^38^. Compared with previously-reported microvalve-based single cell RT-qPCR system, the present droplet array system has a relatively higher detection limit of gene copies, which may limit its applications in the analysis of low-expression RNAs in individual cells. Since we have demonstrated the high detection limit was mainly attributed to the inhibition of suspension buffer and cell lysate, the detection sensitivity could be improved by incorporating gene purification steps to remove interfering species after cell lysis^39^ or using PCR reagents that are more tolerating reaction inhibition^15^.

## Methods

### Chemicals and materials

All solvents and chemicals were used as received unless stated otherwise. TaqMan microRNA RT kit (No. 4366596), TaqMan universal PCR master mix (AmpErase UNG, No. 4324018) and primers (No. 4395356) were purchased from Applied Biosystems (Orbital Instrument Co., Shanghai, China). The sequence of mature mir-122 (UGGAGUGUGACAAUGGUGUUUG) selected from the Sanger Center miRBase at http://microrna.sanger.ac.uk/sequences and synthetic mature mir-122 oligonucleotides were synthesized by GenePharma (Shanghai, China). DEPC-treated water (TaKaRa, Dalian, China) was used throughout to prepare the PCR samples and reagents. RNase Remover (TianDZ Gene Tech. Beijing, China), prepared in water or ethanol (2%, v/v), was used to wash microchips and capillaries. Mineral oil, Span 80, bovine serum albumin (BSA) and octadecyltrichlorosilane (OTCS) were products of Sigma-Aldrich (St. Louis, USA). Huh-7 cells purchased from CCTCC (Wuhan, China) were cultured in DMEM containing 10% FBS (Gibco, Life Technologies, Carlsbad, USA). Calcein-AM, purchased from Invitrogen (Life Technologies, Carlsbad, USA), was used to fluorescent staining and counting of viable cells in droplets.

### Building of the single-cell RT-qPCR System

The single-cell RT-qPCR system was built by combing a microfluidic robot^28^ for droplet manipulation and real-time fluorescence PCR system^40^ for detection. Briefly, the microfluidic robot is composed of a tapered capillary probe (250 µm i.d., 300 µm o.d. for capillary; 60 µm i.d. and 80 µm o.d. for tip size), a syringe pump (PHD 2000, Harvard Apparatus, Holliston, USA) with a 10 µL gas-tight syringe (1700 series, Hamilton, Reno, USA), an automated *x-y-z* translation stage (Zolix, Beijing, China), and a silicon microchip with hydrophilic spots. The silicon chip was fabricated using previously-developed procedures^27^ including thermal oxidation, hydrophobic silanization with OTCS, spin-coating of positive photoresist, photolithography, and finally wetting etching to generate hydrophilic spot array. The diameter of each hydrophilic spot was 200 μm and the center-to-center distance between adjacent spots was 800 μm. The operation of syringe pump and the moving of *x-y-z* translation stage were synchronously controlled with a program written with Labview (Version 8.0, National Instruments, USA). The real-time fluorescence PCR system consisted of a commercial thermal cycler with a flatted aluminum plate (MGL96G/Y, LongGene, Hangzhou, China), two blue LED lamps (460 nm, 3W, CREE, Durham, USA), a high sensitive CCD camera (DH-SV1401FC/FM, Daheng Image, Beijing, China) with a camera lens (Computar MLM-3XMP, Daheng Image, Beijing, China), and a filter set (470DF35 for LED excitation and 535AF40 for fluorescence emission, Omega Optical, Brattleboro, USA) for purifying excitation and fluorescence light. A Labview program was used to switch on the LED lamps and record the fluorescent images of droplet array chip periodically at the annealing step of PCR thermocycling with the CCD camera. The fluorescence intensity of each droplet was automatically read from the droplet images with a Labview program and processed with Origin (Version 7.5, OriginLab Corporation, Northampton, USA)^27^.

### Quantification of mir-122 in single cell with two-step RT-qPCR

Before single cell analysis, silicon chip and capillary probe were sequentially washed with RNase-free ethanol, 2% RNase Remover, and DEPC-treated water. Huh-7 cells were stained with Calcein-AM in cell culture media (0.5‰, v/v) and then washed with PBS buffer for three times. After counting and dilution, the cell suspension was loaded in a PCR tube mounted on the *x-y-z* translation stage. The silicon chip was also mounted on the translation stage and covered with a layer of mineral oil (1 mm thickness). The *x-y-z* translation stage was set with an initial velocity of 10 mm/s, an acceleration of 40 mm/s^2^, and a uniform velocity of 40 mm/s. The syringe and capillary were filled with degassed water and the capillary tip was filled with 50-nL mineral oil containing 0.5% Span 80 (v/v) to separate the aspirated sample/reagent solution from the carrier water in the capillary.

The procedures for multi-step single cell RT-qPCR assay are illustrated in Figure 1. The droplet array containing cells was generated by aspirating 100 nL cell suspension into the capillary, and then sequentially printing droplets on hydrophilic spots of the chip with a volume of 2 nL for each droplet using the coordination of switching of the syringe pump and moving of the *x-y-z* translation stage (Figure 1a). The syringe pump was operated at a relative lower flow rate of 300 nL/min to ensure the volume precision of droplet generation. The cell number in each droplet was counted using a fluorescent stereomicroscope (SMZ 1500, Nikon, Japan) and indexed as the two dimensional (2D) spatial information of the droplet array. The cells in droplets were then thermally lysed under a temperature of 95°C for 5 min.

The microchip was remounted on the *x-y-z* translation stage. To ensure the microchip to be mounted at the same position, a CCD camera was installed on top of the translation stage to help to precisely align the position of the microchip. The microRNA RT mix (total volume of 10 μL containing 0.1 μL dNTP mix, 0.7 μL Multiscribe™ RT enzyme, 1 μL 10 × RT Buffer, 0.13 μL RNase Inhibitor, 2 μL 5 × RT primers, 0.5 μL 1 mg/mL BSA, and 5.57 μL DEPC-treated water) was sequentially dispensed into droplets with a volume of 18 nL/droplet as shown in Figure 1b. The dispensed RT reagent could easily merge with the preformed droplets while contacting with each other. Reverse transcription of mirRNA into cDNA was performed by heating the microchip to 16°C for 30 min, 42°C for 30 min and 85°C for 5 min. For PCR amplification, PCR reagent was freshly prepared by mixing 10 μL 2 × TaqMan universal PCR master mix, 1 μL 20 × TaqMan probe and primers, 0.4 μL DEPC-treated water and 0.6 μL 1 mg/mL BSA to prevent enzyme absorption on droplet/oil interface. Similarly, the PCR reagent was also sequentially dispensed into droplets with a volume of 30 nL/droplet (Figure 1c). Thus, the final volume of each droplet was 50 nL. In both dispensing steps for RT and PCR reagent additions, the syringe pump was set at a relatively higher flow rate of 800 nL/min to increase droplet processing throughput. Finally, the microchip was placed on the thermal cycler and heated at 95°C for 5 min, followed by 45 cycles of 95°C for 30 s, and 60°C for 70 s (Figure 1d).

## Author Contributions

Y.Z. and Y.X.Z. have the equal contribution to this work. Y.Z. built both the droplet manipulation platform and real-time fluorescence detection platform. Y.Z., Y.X.Z. and W.W.L. fabricated microchips and performed the experiments. Y.M. cultured the Huh-7 cells. Y.Z., Y.X.Z., Q.F. and B.Y. processed the data and drafted the manuscript. Q.F. and B.Y. conceived the project and led the research process. All authors discussed the results and approved on the manuscript.

## Supplementary Material

Supplementary Information Supplementary Information

## Figures and Tables

**Table 1:** The effect of PBS concentration on mir-122 quantification, calculated from the standard curves in Figure 4

PBS concentration	Linear range (copies/droplet)	PCR efficiency	Limit of detection (copies/droplet)
**50 mM**	10^6^–10^8^	49.99%	1000000
**10 mM**	2 × 10^5^–2 × 10^8^	80.47%	200000
**2 mM**	3 × 10^3^–2 × 10^6^	86.96%	3000
**0.5 mM**	1.2 × 10^2^–2 × 10^6^	90.94%	120
**0 mM**	6–2 × 10^7^	98.03%	6
