# Supplementary material for: Printing 2-Dimentional Droplet Array for Single-Cell Reverse Transcription Quantitative PCR Assay with a Microfluidic Robot
Source: Sci Rep. 2015 Apr 1;5:9551. doi: 10.1038/srep09551 (PMC4381353; doi:10.1038/srep09551)
Supplement: Supplementary Information [file srep09551-s1.doc]

**SUPPORTING INFORMATION FOR**

Printing a 2-dimentional droplet array for single-cell reverse transcription quantitative PCR assay with a microfluidic robot

Ying Zhu,‡ Yun-Xia Zhang,‡ Wen-Wen Liu, Yan Ma, Qun Fang,* and Bo Yao*

Institute of Microanalytical Systems, Department of Chemistry, Zhejiang University, Hangzhou, 310058, China

‡ These authors contributed equally to this work.

* To whom correspondence should be addressed. Email: [fangqun@zju.edu.cn](mailto:fangqun@zju.edu.cn); [yaobo08@zju.edu.cn](mailto:yaobo08@zju.edu.cn)

Figure S1. Evaluation of the cross contamination between alternatively generated droplets containing mir-122 of 9.6×10^8^ copies/droplet and DEPC-treated water with and without the washing steps. The working standard curve was *y = –3.61x + 35.53*. Without the washing steps, the average concentration of mir-122 in the contaminated DEPC-treated water droplets was calculated as 2.12×10^5^ ± 1.8×10^5^ copies/droplet (n=3). With the washing steps, the average concentration of mir-122 in the contaminated droplets was 55 ± 23 copies/droplet (n=3).

Figure S2. Investigation of the effect of PBS concentrations on the microRNA integrity during cell lysis process by lysing cells in two different PBS buffers (0.2× and 1×) and then measuring their Ct values. The PCR amplification curves indicating the mir-122 quantities were obtained by lysing cells in 0.2× (a) and 1× (b) PBS buffers, respectively. The average Ct values were calculated as 24.08 ± 0.13 (n=10) and 24.19 ± 0.19 (n=12), respectively.


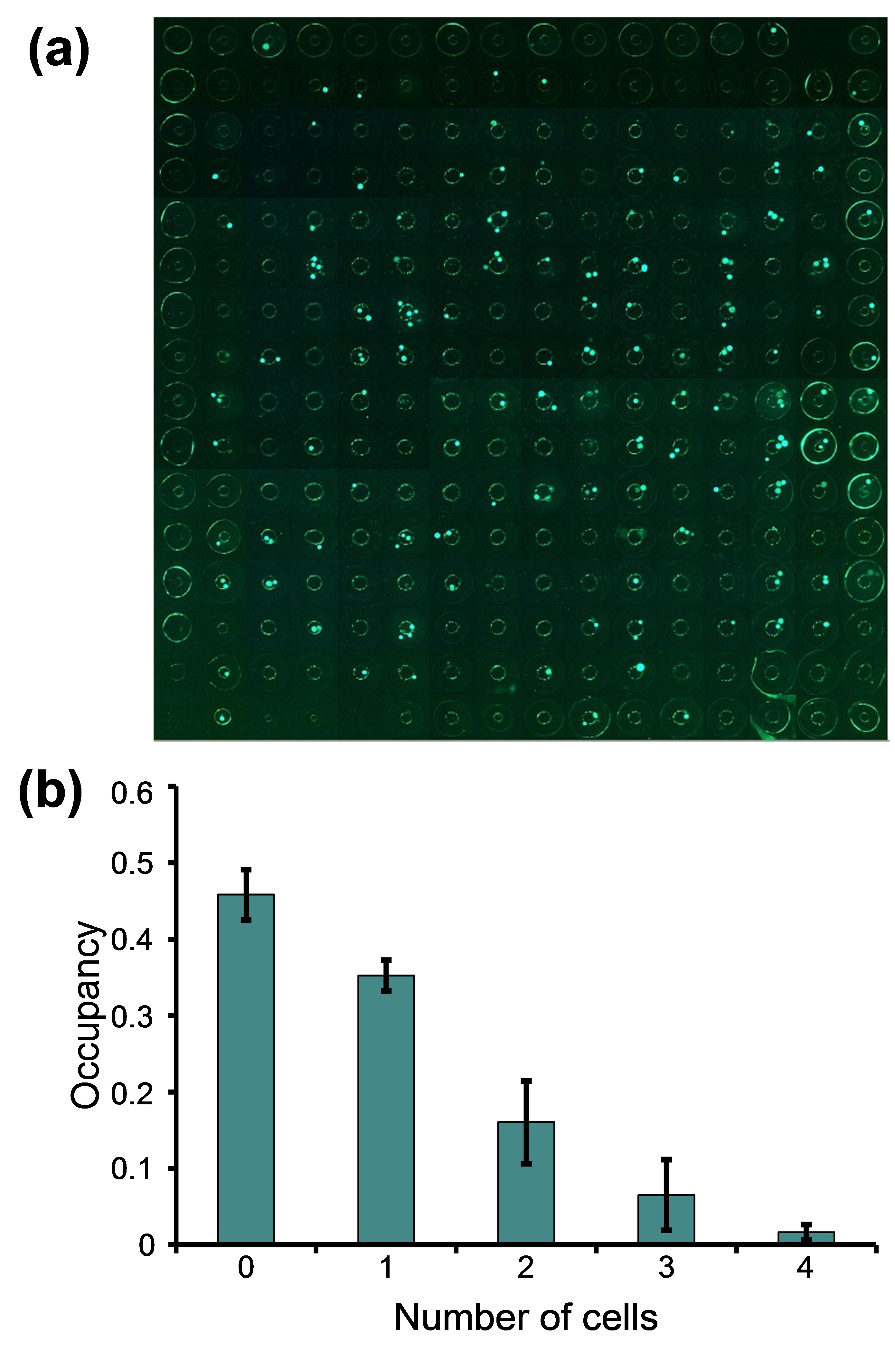


Figure S3. (a) Assembled fluorescence image showing an array of droplets containing cells. Cells were stained with Calcein-AM to facilitate cell identification and counting. The volume of each droplet is 2 nL. (b) Probability distribution of different cell number per droplet.
